# Treatment of isolated gingival recession by tunneled coronally advanced flap and orthodontics: A case report

**DOI:** 10.34172/japid.026.3900

**Published:** 2026-03-24

**Authors:** Alejandro Guillen, Francisco Wilker Mustafa Gomes Muniz, Andrea Vergara-Buenaventura

**Affiliations:** ^1^Department of Periodontology, Faculty of Health Sciences, Universidad Peruana de Ciencias Aplicadas, Lima, Peru; ^2^Department of Periodontology, Graduate Program in Dentistry, Federal University of Pelotas, Pelotas, Brazil

**Keywords:** Connective tissue graft, Gingival phenotype, Gingival recession, Orthodontic treatment, Periodontal plastic surgery

## Abstract

Gingival recession (GR) in malpositioned teeth with a thin periodontal phenotype poses a high risk during orthodontic movement. Phenotype modification before orthodontic treatment may reduce complications and improve clinical outcomes. A 27-year-old woman presented with a 3-mm RT2 gingival recession and<1 mm of keratinized tissue (KT) in tooth 31, which was labially displaced. Cone-beam computed tomography (CBCT) revealed the absence of the vestibular bone, and clinical evaluation confirmed a thin periodontal phenotype. To prevent GR progression during orthodontic movement, a tunneled coronally advanced flap (TCAF) combined with a subepithelial connective tissue graft (SCTG) was performed. Three months later, orthodontic treatment was initiated to lingualize the incisor. At 3-, 6-, and 10-month follow-ups, clinical improvements included an increase in KT width from<1 mm to 5 mm and greater gingival thickness. Complete root coverage was achieved and remained stable after orthodontic therapy. Esthetic results were rated as excellent using the root coverage esthetic score (RES=10/10). The patient reported minimal postoperative discomfort and no complications. This case highlights the successful use of TCAF with SCTG to modify the phenotype before orthodontic treatment in a high-risk GR case. The interdisciplinary approach led to favorable soft tissue augmentation, complete root coverage, and esthetic stability, supporting its use in similar clinical scenarios.

## Graphical Abstract

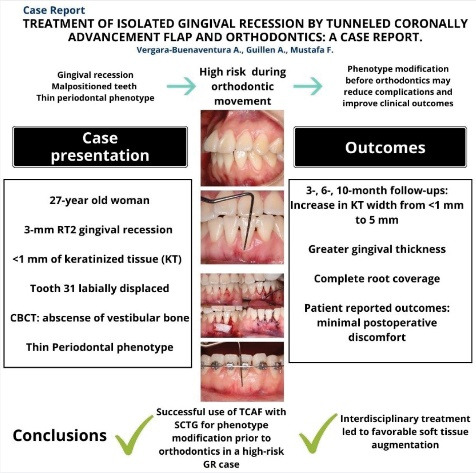

## Introduction

 The apical migration of the gingival margin beyond the cementoenamel junction (CEJ), also known as gingival recession (GR),^1^ is a multifactorial mucogingival condition with esthetic implications that can lead to dentin hypersensitivity and root caries.^2^ GR has been proposed to be associated with several clinical and anatomic components that indicate the need for surgical intervention. However, it has been suggested that the absence of treatment increases the risk of GR progression.^2,3^

 In more challenging cases, such as when a GR is found in malpositioned teeth with bony dehiscence or fenestration, orthodontic treatment (OT) is suggested to reposition the tooth within the bone housing.^4^ A previous evaluation of anatomical conditions, such as periodontal phenotype (keratinized tissue [KT] and gingival thickness [GT]) and tooth position, should be considered in periodontal interdisciplinary treatment planning.^2^ It is known that significant facial GR in the presence of a thin periodontal phenotype may contraindicate the orthodontic tooth movement without adjunctive periodontal treatment.^5^

 Patients with a thin periodontal phenotype, characterized by reduced GT and narrow KT, are particularly susceptible to soft tissue damage during orthodontic movement.^6^ In these cases, labial or buccal movement of the tooth may result in dehiscence or fenestration of the alveolar bone, leading to GR or worsening of preexisting defects.^5^

 Periodontal phenotype modification therapy (PhMT) includes procedures such as hard tissue augmentation (PhMT-b) and soft tissue augmentation (PhMT-s) to modify the periodontal phenotype so that it can withstand orthodontic forces.^6^ PhMT-s includes histologic and clinical changes that involve thickening the epithelial layer and increasing the number and density of collagen fibers in the lamina propria with soft tissue grafts.^7,8^ The resulting clinical increase in KT width to 2 mm and in GT to 1 mm provides stability to the gingival margin.^4^

 Mucogingival interceptive surgery is effective in maintaining KT around flared teeth, improving patient comfort, and preventing the onset or progression of GR.^8,9^ Some reports suggest that mucogingival surgery should ideally be performed after the OT is completed. However, when the patient has insufficient KT and a thin phenotype, early intervention may be justified.^9^ Among the numerous root coverage procedures, the tunneled coronally advanced flap (TCAF)^10^ is a minimally invasive surgical technique. This approach aims to enhance flap blood supply and graft vascularization, improving clinical, esthetic, and patient-reported outcomes of RT2 GRs with deficient papilla.

 Although the TCAF technique is relatively new and supported by limited clinical evidence, recent studies have reported favorable outcomes around both teeth and dental implants.^11^ In the context of GR, its combination with autogenous grafts or biomaterials such as porcine-derived acellular dermal matrix has achieved up to 89% root coverage, volumetric soft tissue gain, and high patient satisfaction without complications.^12^ Additional reports highlight low postoperative pain and minimal analgesic use,^13^ as well as successful integration with regenerative materials, such as platelet-derived growth factor-BB–enriched collagen matrices, resulting in soft tissue thickening and reduced bone dehiscence.^14^ TCAF has also shown promising results in complex cases, including RT3 defects and anatomically challenging sites, particularly when integrated into staged approaches following phenotype modification.^15,16^

 Given the limited long-term evidence and the potential advantages of the TCAF technique in high-risk periodontal scenarios, documenting its application in real-world clinical settings is valuable. The objective of this case report is to present the interdisciplinary management of an RT2 GR in a labially displaced lower incisor with a thin periodontal phenotype, using the TCAF technique combined with an SCTG as a phenotype modification strategy before OT. This case contributes to the growing body of literature supporting the use of TCAF in high-risk periodontal scenarios, where early soft tissue intervention may optimize clinical and esthetic outcomes.

## Case Report

 The present case report was carried out following the recommendations of the Case Report (CARE) guidelinesand in accordance with the Declaration of Helsinki.

###  Clinical Presentation 

 A 27-year-old female with no systemic conditions and no contributory medical or dental history was referred to the Periodontics Department of the Universidad Peruana de Ciencias Aplicadas following orthodontic evaluation. Periodontal examination showed a stable periodontal condition, with no periodontal pockets, a bleeding-on-probing score of 28%, and an O’Leary plaque score of 54%. A 3-mm GR with a narrow band ( < 1 mm) of KT was observed in the lower left central incisor (tooth #31). All measurements were performed using a UNC-15 periodontal probe (Hu-Friedy®, USA) by an experienced dentist.

**Figure 1:**
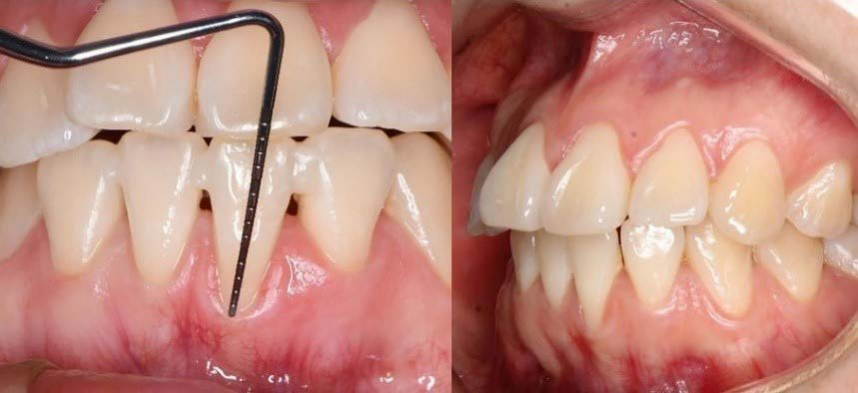
Initial condition

 The mandibular anterior region presented with a thin periodontal phenotype and labial displacement of tooth #31, with evident root prominence, suggesting a potential alveolar bone defect (Figure 1). According to the most recent classification, the GR was classified as RT2 A- (detectable CEJ and absence of cervical step).^1,2^

**Figure 2:**
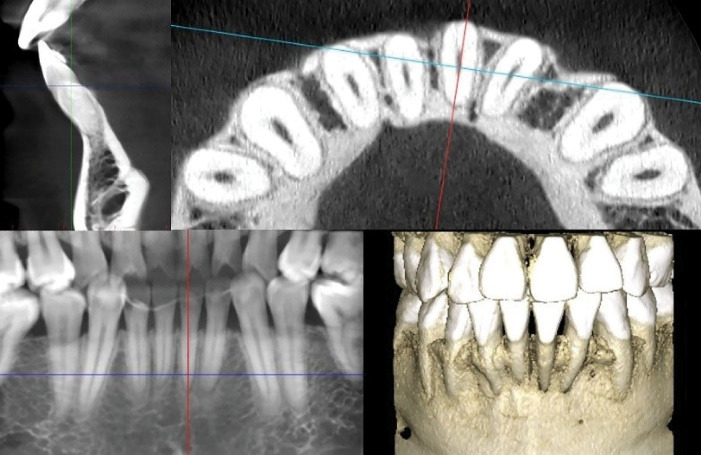
Initial CBCT

 Gingival phenotype was assessed clinically and confirmed via cone-beam computed tomography (CBCT) performed with a lip retractor to improve visualization. CBCT analysis showed the absence of the vestibular bone plate and a thin soft tissue profile (Figure 2). Based on the clinical and radiographic findings, the patient was considered at high risk for further periodontal breakdown during orthodontic movement. Therefore, phenotype modification with mucogingival surgery was indicated before OT.

###  Case Management

 After explaining the surgical and orthodontic plan, the patient signed the informed consent forms required by the University. A full-mouth prophylaxis was performed 7 days before surgery.

###  Periodontal Phenotype Modification Therapy 

 On the day of surgery, an improved periodontal condition was observed. Local anesthesia was administered using 2% lidocaine with epinephrine 1:100,000. A TCAF was performed according to the technique described by Barootchi and Tavelli,^10^ using a Swann-Morton steel mini-blade. A single vertical releasing incision was made, followed by the elevation of a split-thickness trapezoidal surgical papilla, measuring approximately 1 mm longer than the recession defect.

 Vestibular muscle detachment and internal frenectomy were carried out to reduce flap tension, allowing passive coronal advancement of the flap by approximately 2 mm beyond the CEJ. Root planing and decontamination were performed mechanically with Gracey curettes.

 A subepithelial SCTG was harvested from the palatal donor site using a free gingival graft technique, followed by extraoral deepithelialization. The graft was trimmed and inserted into the tunnel beneath the non-incised papillae using microinstruments, ensuring full passive adaptation without tension.

**Figure 3:**
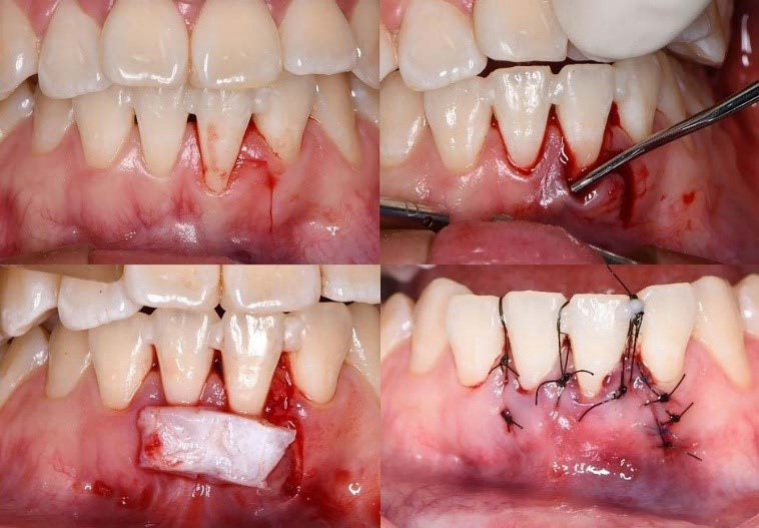
Surgical protocol

 The graft was stabilized using 5-0 nylon simple interrupted sutures, and double cross sutures were applied to the papillae for additional stabilization. Temporary splinting with flowable light-cured resin was used to reinforce flap position during initial healing, following the method described by Zuhr et al.^17^ (Figure 3). The palatal donor site was treated with a collagen hemostatic sponge, secured with X-sutures, and protected with flowable resin according to Meza-Mauricio et al.^18^

###  Postoperative Care and Monitoring

 The patient received oral and written postoperative instructions, and the following medications were prescribed: amoxicillin 500 mg (three times daily for 7 days), ibuprofen 600 mg (three times daily for 3 days), and 0.12% chlorhexidine gluconate rinse (twice daily for 14 days). Toothbrushing at the surgical site was avoided for 14 days.

 Follow-ups evaluations were conducted 3, 7, 14, and 30 days after surgery, with no complications such as dehiscence, graft exposure, or suture loss. Postoperative complications were monitored during all follow-up visits through clinical examination. The patient reported moderate edema during the first postoperative week and mild pain, which resolved spontaneously by day 7. The sutures were removed on day 14.

###  Orthodontic Phase

 Orthodontic treatment (OT) began 3 months after mucogingival surgery and was conducted by a single orthodontist. Fixed appliances were placed using standard edgewise brackets. The lingualization of tooth #31 was achieved using continuous archwire mechanics with a 0.016 × 0.022” stainless steel wire. Light orthodontic forces were applied to minimize periodontal trauma. Adjustments were performed every 4 weeks, and the orthodontic phase is still ongoing at the time of this report.

Table 1 summarizes the clinical timeline, including interventions and follow-up visits.

**Table 1:** Timeline summarizing key interventions and Follow-Up assessments

**Time interval**	**Clinical step/intervention**
Day 7	Full-mouth prophylaxis
Day 0	TCAF surgery with SCTG (phenotype modification surgery)
Day 3	Postoperative control (healing evaluation, pain/edema monitoring)
Day 7	Postoperative control (continued healing assessment)
Day 14	Final postoperative check and suture removal
Day 30	Evaluation of soft tissue changes (increased GT and KT observed)
Month 3	Clinical re-evaluation (Figure 5) and start of orthodontic treatment (fixed appliance, archwire mechanics initiated)
Month 6	Clinical re-evaluation (Figure 6)
Month 9	Final follow-up assessment (root coverage stability, esthetic outcome – Figure 7)

###  Surgical Outcomes

**Figure 4:**
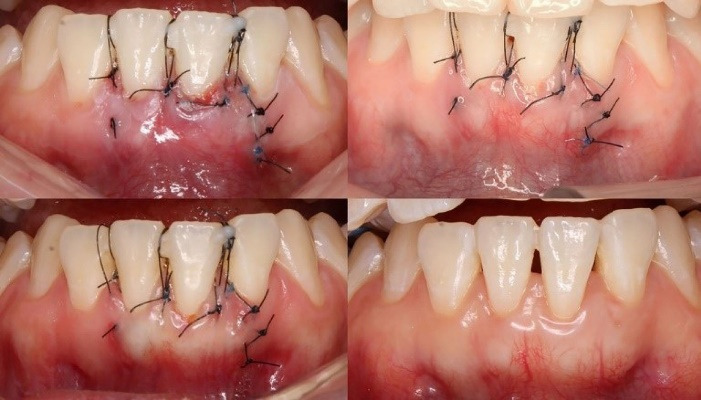
Follow-up (3, 7, 14, and 30 days after surgery)

 Periodontal outcomes were evaluated at 1, 3, 6, and 10 months. At 1 month, a notable increase in GT and keratinized TW was observed (Figure 4).

**Figure 5:**
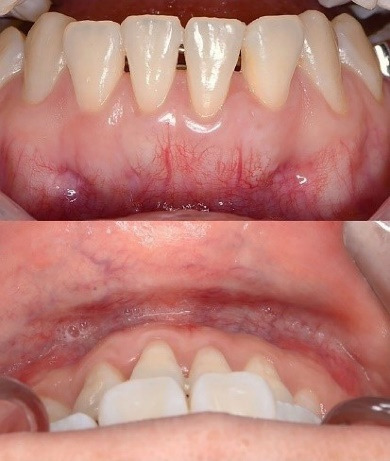
Three-month follow-up

**Figure 6:**
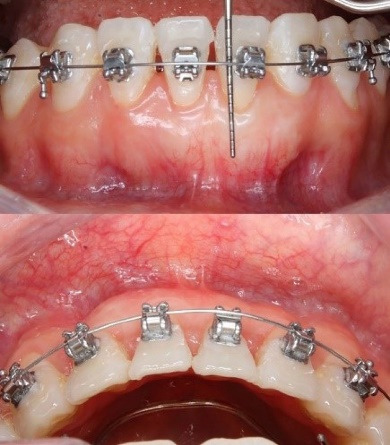
Six-month follow-up

**Figure 7:**
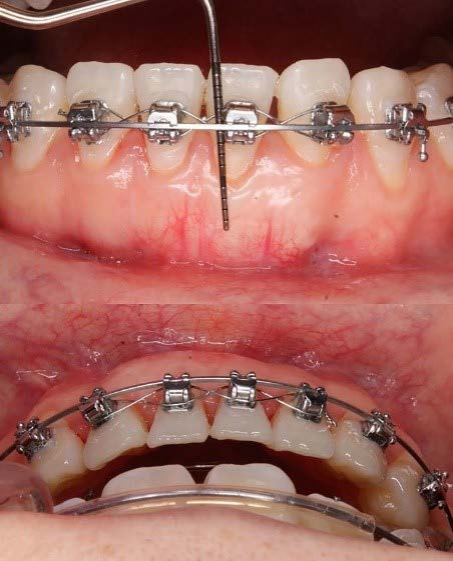
Ten-month follow-up

 At 3 months, labial GT increased visibly, indicating early soft tissue maturation (Figure 5). At 6 months, the apicocoronal dimension of KT increased by 4 mm, and GT also increased, as measured clinically using a UNC-15 periodontal probe (Figure 6). GR was fully resolved in tooth #31, resulting in complete root coverage, which was maintained at 10 months. In this follow-up period, stability of the gingival margin, KT width, and GT was observed during active orthodontic movement (Figure 7). These outcomes reflect both qualitative improvements (complete root coverage, increased soft tissue volume, esthetic integration, and quantitative gains in KT (from < 1 mm to ~5 mm) and GT.

 An aesthetic evaluation was performed at 10 months using the RES19 proposed by Cairo et al.^19^ The assessment was conducted by an experienced periodontist who was not involved in the surgical procedure to ensure objectivity. The treated site achieved a full score of 10/10, with perfect alignment of the gingival margin, intact papillae, and full integration in color and texture with adjacent tissues.

###  Orthodontic Outcomes

 After 3 months of active OT, tooth #31 was successfully lingualized into the alveolar housing using continuous arch mechanics (Figure 6). This movement resulted in an additional improvement in vestibular tissue thickness and enhanced the gingival margin’s coronal position. At the 6- and 10-month follow-up visits, complete root coverage was achieved and maintained, with stable soft tissue contours and no signs of relapse. The periodontal parameters (KTW and GT) remained stable during orthodontic movement.

###  Patient-reported Outcomes 

 In addition to clinical improvements, the patient reported high satisfaction with the esthetic outcome and improved comfort during daily oral hygiene practices. No postoperative sensitivity or discomfort was reported throughout the follow-up period.

## Discussion

 Cases of GR with soft/hard tissue involvement and tooth malposition are challenging situations that need interdisciplinary management. It should include mucogingival surgery and OT to reposition the tooth within its bony housing.^4^ The timing of mucogingival surgery in orthodontic patients is a key consideration for optimal periodontal health and treatment outcomes. However, there is still no consensus on the best time for the mucogingival approach.^20^ Some authors have suggested that mucogingival surgery is ideally performed after the OT is completed or before if there is a high risk of additional recession during OT. This case report describes the efficacy of interdisciplinary therapy for GR using PhMT-s before OT.

 Patients with a thin periodontal phenotype may warrant the use of PhMT with hard or soft tissue grafts to optimize pre-OT clinical conditions.^6^ Clinical and tomographic evaluations showed that the patient presented GR, a thin phenotype, and insufficient KT. The American Academy of Periodontology recommends performing gingival augmentation on teeth with < 2 mm of KT and when significant labial tooth movement is planned with OT.^8^

 Treating mucogingival deficiencies constituted a predictable and efficient modality to increase the apicocoronal dimension of KT, particularly before OT in areas with < 2 mm of KT.^6,9^ Furthermore, this approach helps avoid potential periodontal complications or worsening of preexisting mucogingival defects.^21^

 The decision for a mucogingival approach before OT was confirmed because the direction of orthodontic movement can modify the width of the KT and contribute to the GR progression.^2,9^

 Biologically, enhancing the soft tissue phenotype before orthodontic movement may reduce the risk of further GR by increasing tissue thickness and resistance to mechanical stress. Thicker gingival tissues are less prone to apical migration during labial tooth movement, and sufficient KT provides greater protection against inflammation and trauma during orthodontic mechanics. This improved resilience supports both short- and long-term periodontal stability during and after tooth movement.^6,8,9^

 When a patient presents vestibular GR and a thin periodontal phenotype, significant tooth movement without adjunctive periodontal treatment may be contraindicated.^5^ It is necessary to increase the apicocoronal dimension of KT before applying orthodontic forces.^6^

 Previous studies have suggested that soft tissue augmentation before OT may help prevent GR in high-risk patients.^6^ The patient presented an RT2 GR, and the papillary involvement led to a minimally invasive approach.^2^ For this purpose, it was decided to use a TCAF^10^ SCTG. The TCAF technique combines the advantages of the coronally advanced flap (CAF) and the tunnel technique, with minimal morbidity, rapid healing, and satisfactory esthetics.^10^ Conventional CAF may offer higher rates of root coverage but requires greater flap manipulation, which may compromise the blood supply and affect healing.^22^ In contrast, the TCAF approach has the potential to enhance flap blood supply and graft vascularization, improving clinical and aesthetic outcomes.^10^

 This case report involved a single RT2 GR defect, in which significant gains in GT and root coverage were achieved following the staged surgical‒orthodontic approach. These clinical outcomes are in line with those reported by Barootchi et al.,^12^ who evaluated the combination of TCAF with a porcine-derived acellular dermal matrix for multiple GRs and achieved 89% mean root coverage with significant volumetric gain and patient satisfaction. From the patient’s perspective, the outcomes were highly satisfactory. The patient reported no postoperative complications, discomfort, or esthetic concerns during follow-up, suggesting that the TCAF approach may offer favorable recovery, regardless of the graft material used. Likewise, a case report by Mancini and Mancini^13^ showed promising patient-reported outcomes after TCAF, with minimal pain and analgesic use.

 Our clinical report showed a marked increase in both the width and thickness of KT, contributing to a favorable modification of the gingival phenotype before orthodontic movement. This strategy aligns with the staged approach proposed by Watanabe et al.,^15^ who treated multiple RT3 GRs by first enhancing soft tissue phenotype and later applying the TCAF technique, achieving significant clinical attachment gain and long-term tissue stability. Similarly, Lin et al.^16^ demonstrated that, in anatomically complex cases, combining TCAF with soft tissue augmentation techniques enhances flap integrity and promotes soft tissue stability at follow-up.

 The interdisciplinary treatment resulted in an increased apicocoronal KT dimension and improved GT. In addition, the subsequent lingualization of the lower incisors contributed to a gain in vestibular tissue thickness. Although complete root coverage was not the primary outcome of the surgery, the final result after OT was esthetically and functionally successful.

 This report is inherently limited by its design as a single case study, which limits the generalizability of its findings. Additionally, although the 10-month follow-up showed favorable clinical stability, longer-term data are needed to confirm the durability of the results. Further prospective studies with larger sample sizes and extended follow-up periods are needed to validate the clinical benefits of pre-orthodontic phenotype modification in similar high-risk cases.

## Conclusion

 This case highlights the clinical benefits of applying a staged interdisciplinary approach involving soft tissue phenotype modification before orthodontic movement in patients with a thin periodontal phenotype and RT2 GR. The use of the TCAF technique, combined with SCTG, led to increased KT width and GT, and to stable root coverage during and after OT. These outcomes support the rationale for early mucogingival intervention in high-risk cases to enhance periodontal resilience and minimize complications. Clinicians should consider phenotype modification as a strategic step before initiating orthodontic forces, particularly in anatomically compromised or labially displaced teeth.

## Competing Interests

 The authors have no financial interest in the companies whose materials are included in this article. The authors declare that they have no competing interests regarding authorship and/or publications of this paper.

## Consent for Publication

 Written informed consent was obtained from the patient to authorize the research. Informed consent was obtained from the participant.

## Data Availability

 The data supporting the findings of this study are available from the corresponding author upon reasonable request.

## Ethical Approval

 Not Applicable.
